# Comparison of Oxidative Stress Parameters in Heart Failure Patients Depending on Ischaemic or Nonischaemic Aetiology

**DOI:** 10.1155/2019/7156038

**Published:** 2019-09-17

**Authors:** Ewa Romuk, Celina Wojciechowska, Wojciech Jacheć, Jolanta Nowak, Jacek Niedziela, Jolanta Malinowska-Borowska, Anna Głogowska-Gruszka, Ewa Birkner, Piotr Rozentryt

**Affiliations:** ^1^Department of Biochemistry, School of Medicine with the Division of Dentistry, Medical University of Silesia, Jordana 19 Street, 41-808 Zabrze, Poland; ^2^Second Department of Cardiology, School of Medicine with the Division of Dentistry, Medical University of Silesia, M. C. Skłodowskiej 10 Street, 41-800 Zabrze, Poland; ^3^3rd Department of Cardiology, SMDZ in Zabrze, Medical University of Silesia, Silesian Centre for Heart Disease, 41-800 Zabrze, Poland; ^4^Department of Toxicology and Health Protection, School of Public Health, Medical University of Silesia, 41-902 Bytom, Poland

## Abstract

**Background:**

Abnormalities in the oxidative and antioxidant states causing oxidative stress were both found in heart failure (HF) of various aetiologies and atherosclerosis.

**Aim of Study:**

The goals of the study were as follows: comparison of oxidative stress parameters (OSP) in ischaemic cardiomyopathy (ICM) (*n* = 479) and nonischaemic cardiomyopathy (nICM) (*n* = 295) patients; assessment of the relationships of OSP with functional capacity (NYHA class), maximal oxygen consumption (max.O2), left ventricle ejection fraction (LVEF), and NT-proBNP concentration; and determination of the mutual relations of OSP in subgroups of patients with ICM and n-ICM.

**Methods:**

Serum concentrations of total antioxidant capacity (TAC), total oxidant status (TOS), uric acid (UA), bilirubin, albumin, protein sulfhydryl groups (PSH), and malondialdehyde (MDA) were measured. The oxidative stress index (OSI) and MDA/PSH ratio were calculated.

**Results:**

Higher concentrations of TAC (1.14 vs 1.11 mmol/l; *p* < 0.001) and MDA (1.80 vs 1.70 *μ*mol/l; *p* < 0.05) and higher MDA/PSH ratios (0.435 vs 0.358; *p* < 0,001) were observed in ICM than in nICM patients. Simultaneously, lower values of the OSI index (4.27 vs 4.6; *p* < 0, 05), PSH (4.10 vs 4.75 *μ*mol/g of protein; *p* < 0,001), and bilirubin (12.70 vs 15.40 *μ*mol/l; *p* < 0,001) concentrations were indicated in ICM patients. There were no differences in TOS, UA, and albumin between the examined groups. The NYHA class and VO2max correlate with MDA, bilirubin, and albumin in both groups, while with UA only in the ICM group. Correlations between the NYHA class, VO2max, and PSH were indicated in nICM. The association of LVEF with UA, bilirubin, and albumin has been demonstrated in the ICM group. The study showed negative correlations between TAC, MDA, and PSH and positive between TAC and MDA in both groups. In ICM patients, MDA positively correlated with UA. A negative correlation between PSH and concentrations of UA and bilirubin was expressed only in the nICM group.

**Conclusion:**

The obtained results confirm the relationship between the severity of HF and oxidative stress. The mechanisms of oxidative stress and antioxidant defence are partially different in the ICM and the nICM patients.

## 1. Introduction

Heart failure (HF) with reduced ejection fraction is a progressive, systemic disease resulting from systolic dysfunction, an often enlarged left ventricle, with low, insufficient cardiac output to meet tissue demands. In many cases, reduced myocardial contractility is a result of ischaemic heart disease associated with the other systemic process of atherosclerosis [1].

Abnormalities in the oxidative and antioxidant states causing oxidative stress were found in heart failure of various aetiologies. Furthermore, increasing experimental evidence supports the concept that oxidative stress is increased in the failing heart and contributes to the pathogenesis of myocardial remodelling [2].

Systolic heart dysfunction leads to tissue hypoxia. In these conditions, we observed an imbalance in mitochondrial electron transport, which leads to increased oxidative stress, intracellular Ca^2+^ overload, and cell death. The formation of reactive oxygen species (ROS) in the heart and other tissues occurs as a result of several mechanisms. They can be produced by xanthine oxidase (XO), NAD(P)H oxidases, and cytochrome P450, by autoxidation of catecholamines and by the uncoupling of NO synthase (NOS) [3, 4]. Oxidative stress appears as a result of an imbalance between free radical production and endogenous antioxidant defences [5].

One of the methods to detect oxidative stress is a measurement of organic molecules that are products of harmful ROS effects on the integrity of biological tissue, e.g., malondialdehyde (MDA). MDA arises as a result of the fragmentation of polyunsaturated fatty acids undergoing attack by ROS and is a generally accepted index of lipid peroxidation [6].

Systemic oxidative stress can also be measured as a depletion of free thiol in the serum [7]. Protein thiols predominate in the serum, and as thiols are easily oxidised by ROS, therefore, their concentration may reflect the overall redox status. Sulfhydryl groups of proteins (PSH) and low-molecular weight thiols influence the overall redox balance generating reversible semioxidised species [8].

Both protein modifications and coexisting increased lipid oxidation confirm the existence of systemic oxidative stress in heart failure patients [9]. Besides reflecting the redox status, thiols are active components of total antioxidant capacity (TAC).

Uric acid is considered as an important component of the antioxidant system. It is a final product of the enzymatic degradation of purines, and it is released from hypoxic tissues [10].

Due to the fact that xanthine oxidase produces uric acid (UA) in proportion to the production of ROS, the uric acid would be a very valuable and readily available biomarker of oxidative stress in the cardiovascular system. Serum levels of UA probably present an adaptive response against oxidative stress [11].

Another metabolic transformation, which probably affects the redox status, is heme degradation with reduction of biliverdin to bilirubin by biliverdin reductase, with subsequent oxidation of bilirubin to biliverdin by ROS [12].

Because of the large number of different antioxidants in the serum, we decided to assess the total antioxidant capacity (TAC), total oxidant status (TOS), and TOS/TAC ratio, also called the oxidative stress index (OSI), in heart failure patients depending on ischaemic (ICM) and nonischaemic (nICM) aetiologies [13–15]. Additionally, we estimated PSH, MDA, MDA/PSH ratio, and some nonenzymatic antioxidants (albumin, uric acid, and bilirubin). We assessed the relation between redox parameters and the clinical status in groups of patients with ICM and nICM heart failure origin.

## 2. Study Group and Methods

### 2.1. Patients

In our study, we used data collected in the prospective registry of heart failure (PR-HF), undertaken since 2003, and the SICA-HF (Studies Investigating Comorbidities Aggravating Heart Failure) study described elsewhere [16]. The PR-HF contained data on consecutive, unselected patients (age above 18 years), with HF with reduced left ventricle ejection fraction (LVEF ≤ 40%), diagnosed according to published contemporary ESC criteria and treated for at least 3 months before inclusion, who were referred to our Inpatient Clinic as potential candidates for heart transplantation. Exclusion criteria comprised of infectious disease, liver disease with enzymes more than 4 times higher than normal, connective tissue disease, endocrine disorders, chronic kidney disease, stage 4 and 5 malignancies, active bleeding, alcohol abuse, and known antioxidant supplementation. Patients who were included had to be clinically compensated and received optimal pharmacotherapy. Study criteria were fulfilled in 1216 PR-HF or SICA-HF. Data of 774 participants who had completed the clinical and laboratory assessments were included in the final analysis. For the purpose of this study, we divided patients into two groups: ischaemic (479 patients, 56, 44 ± 8, 04) and nonischaemic (295 patients, 47, 40 ± 11, 77) systolic heart failure.

### 2.2. Clinical Assessments

Noninvasive clinical assessments included physical examination, ECG, and echocardiography. The NYHA classification and cardiopulmonary exercise testing (CPX) were used to assess functional capacity [17]. Cardiopulmonary exercise testing was performed using VMAX—oxygen consumption scanner (General Electric, Milwaukee, USA). After a 5-minute rest period, patients underwent a symptom-limited treadmill exercise test (modified Bruce's protocol). Respiratory gas exchange data, minute ventilation, and oxygen consumption were collected continuously. Peak oxygen consumption was measured as an arithmetic mean of values recorded within the last 30 seconds before cessation of exercise and was expressed in mL/min^∗^kg. History of smoking was defined as current or previous use of tobacco products. Comorbidities such as hypertension, hypercholesterolemia, or diabetes mellitus were recognised based on clinical history, current medication, or actual measurements of the respective variables. Body mass and height were measured on the day of blood sampling using a certified scale (B150L, Redwag, Zawiercie, Poland). By dividing the weight in kilograms by height in meters squared, we calculated the patients' body mass indexes (BMI). Echocardiographic images were acquired in standard views as recommended by the American Society of Echocardiography Committee [18]. The left ventricular end-diastolic volume (EDV) and end-systolic volume (ESV) were obtained from the apical 4- and 2-chamber views by the modified Simpson's method. The left ventricular ejection fraction (LVEF) was calculated in a standard manner as follows: (EDV − ESV) × 100/EDV, to assess ventricular systolic function (Sonos 5000 Hewlett-Packard Ultrasound Scanner; Hewlett-Packard, Andover, MA, USA). All patients underwent coronary angiography within six months before inclusion. Ischaemic cardiomyopathy was determined based on a definition proposed by Felker et al. [19].

### 2.3. Biochemical Methods

Blood samples for laboratory assessments were obtained from the patients at the study inclusion. Serum was separated by centrifugation at 1500 g for 10 minutes and frozen at −70°C. Uric acid, bilirubin, and albumin concentrations were measured using the colorimetric method (Roche, Cobas 6000e501). NT-proBNP was measured with the use of the chemiluminescence method (Roche, Cobas 6000e501). Additionally, we determined lipid parameters, blood haemoglobin, and serum creatinine concentrations using routine techniques. The total oxidant status (TOS) was measured by the spectrophotometric method developed by Erel [13]. In this method, oxidising materials contained in the sample lead to the oxidation of Fe^2+^ ions to form Fe^3+^. The reaction proceeds in an acidic environment and consists of measuring the colour intensity of Fe^3+^ ion complexes with xylenol orange. TOS was expressed in mmol/l. Total antioxidant capacity (TAC) was also measured using the spectrophotometric method developed by Erel [14]. The most widely used colorimetric methods are based on the 2,2-azinobis(3-ethylbenzo-thiazoline-6-sulfonate) (ABTS+) reaction. In this method, a colourless molecule, reduced ABTS, is oxidised to a blue-green ABTS+. After mixing the coloured ABTS+ with any substance that can be oxidised, it is reduced to its original, colourless ABTS form again and the reacted substance is oxidised. TAC was expressed in mmol/l. The oxidative stress index (OSI) was expressed as the combined ratio of the total oxidant status (TOS) to the total antioxidant capacity (TAC) in arbitrary unitsto the following formula: OSI = [(TOS, mmol/l)/(TAS, mmol/l)][20]. The concentration of sulfhydryl groups (PSH) in the serum was determined by the Koster method, using 5,5′-dithiobis (2-nitrobenzoic acid)-DTNB, which is reduced by the sulfhydryl group-containing compounds to give an anionic 5-thio-2-nitrobenzoic acid, which is yellow in colour [21]. The absorbance was measured with a Shimadzu 1700 UV-VIS spectrophotometer at a wavelength of 412 nm against a control sample containing water instead of serum. PSH concentration was shown in *μ*mol/g protein.

Malondialdehyde was measured according to the method described by Ohkawa et al., using the reaction with thiobarbituric acid with spectrofluorimetric detection: excitation 515 nm and emission 552 nm. MDA concentration was calculated from the standard curve, prepared from 1,1,3,3-tetraethoxypropane and expressed in *μ*mol/l [22].

### 2.4. Statistical Analysis

Statistical analysis was performed using STATISTICA 13.1 PL (StatSoft, Poland, Cracow). A normal distribution of all variables was evaluated by the Shapiro-Wilk test. The continuous data was presented as a median with the first and fourth quartiles (because of the abnormal distribution of the data). In the case of a normal distribution, continuous variables were compared using the Mann-Whitney *U* test. Categorical data were presented as absolute numbers and percentages and were compared using the chi-square test with Yates correction.

The statistical correlation between the variables was determined using Spearman's rank correlation coefficient, and a *t*-test was performed to decide whether the observed value of the coefficient is significantly different from zero. Results were considered statistically significant if *p* < 0.05. A lack of statistical significance was presented as NS (nonsignificant).

## 3. Results

### 3.1. Clinical and Laboratory Characteristics

774 patients with reduced ejection fraction heart failure (HFrEF), 479 due to ischaemic cardiomyopathy (ICM) (mean age 56.44 years, 86,8% male), and 295 with nonischaemic cardiomyopathy (mean age 47,40 years, 84,4% male) were enrolled into the final study. The ICM group was significantly older. Comorbidities like arterial hypertension and diabetes were more frequent in ICM group. The mean duration of symptoms was similar in both groups and was around four years (Table 1). The patients displayed typical echocardiographic features of impairment of left ventricle systolic function. LVEF was similar in both groups, but the enlargement of the left ventricle was more expressed in nICM patients.

The majority of patients were treated with *β*-blockers (>97%) and either an ACE (angiotensin-converting enzyme) inhibitor or an ARB (angiotensin receptor blocker) (>96%). Most of the patients also received a MRA (mineralocorticoid receptor inhibitor) (>90%). The proportion of patients receiving the treatment as described above were similar in the two groups. However, loop diuretics, digitalis, and inhibitors of the enzyme xanthine oxidase were used more frequently in nonischaemic patients, while statins were more common in the ischaemic group (Table 1). The laboratory data of patients with ischaemic and nonischaemic cardiomyopathy were presented in Table 1. NT-proBNP was similarly increased in both groups. The concentrations of fasting glucose and creatinine were higher in the ischaemic group, while haemoglobin was lower in the nonischaemic group. Some parameters reflecting oxidative-reduction reaction in patients, stratified by HF aetiology, were presented in Table 2.

### 3.2. Comparison of Oxidative Stress Parameters in Groups of Patients Depending on Heart Failure Aetiology

**Figure 1:**
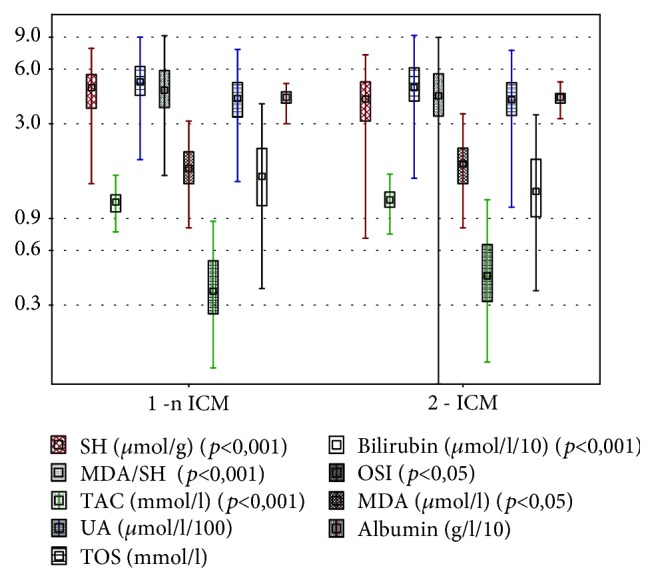
Comparison of oxidative stress parameters in groups of patients depending on heart failure aetiology.

In the ICM patients, we observed a statistically significant higher concentration of TAC (*p* < 0,001) and lower OSI (*p* < 0, 01) than that in the nICM group. There were no differences in TOS concentration between the ICM and nICM groups. Taking into account nonenzymatic oxidative stress parameters, there was a significantly lower bilirubin (*p* < 0,001) concentration in the ICM group in comparison with the nICM patients. There were no differences in uric acid and albumin concentrations between these study groups. PSH concentration was significantly lower (*p* < 0,001) and MDA was significantly higher (*p* < 0, 05) in ICM patients when comparing the data with the nICM group. The MDA/PSH ratio was significantly higher in ICM patients in comparison to the nICM group (*p* < 0,001). Results are shown in Table 2 and Figure 1.

### 3.3. Relationship between Redox Parameters and the Clinical Characteristics, Reflecting the Severity of Heart Failure in Ischaemic and Nonischaemic Cardiomyopathy

#### 3.3.1. ICM

There were significant positive correlations between the NYHA class and TOS (*r* = 0,110; *p* < 0, 05), bilirubin (*r* = −0,269; *p* < 0,001), MDA (*r* = −0,111; *p* < 0, 05), and uric acid (*r* = −0,198; *p* < 0,001).

VO_2_ max correlated inversely with MDA (*r* = −0,129; *p* = 0,011), uric acid (*r* = −0,174; *p* < 0,001), and bilirubin (*r* = −0,262; *p* < 0,001) and positively with albumin (*r* = 0,131; *p* < 0, 01). Similarly, the left ventricle ejection fraction was inversely correlated with uric acid (*r* = −0,176; *p* < 0,001) and bilirubin (*r* = −0,291; *p* = 0,001) concentrations and positively with albumin (*r* = 0,090; *p* = 0, 05), without any correlation with MDA. There was a positive correlation between NT-proBNP with bilirubin (*r* = 0,328; *p* < 0,001) and uric acid (*r* = 0,172; *p* < 0,001), and a negative one with albumin (*r* = −0,279; *p* < 0,001) was observed. Results are shown in Table 3.

#### 3.3.2. nICM

There were significantly positive correlations between the NYHA class and TOS (*r* = 0,156; *p* = 0,008), bilirubin (*r* = 0,244; *p* < 0,001), and MDA (*r* = 0,152; *p* < 0, 05) and inverse correlations with albumin (*r* = −0,275; *p* < 0,001) and PSH (*r* = −0,169; *p* < 0, 01). VO_2_ max correlated negatively with MDA (*r* = −0,188; *p* < 0, 01) and bilirubin (*r* = −0,314; *p* < 0,001). Additionally, VO_2_ max correlated positively with albumin (*r* = 0,144; *p* < 0, 05) and PSH (*r* = 0,179; *p* < 0, 01) concentrations. The left ventricle ejection fraction was inversely correlated only with bilirubin (*r* = −0,134; *p* < 0, 05). Positive correlations between NT-proBNP and bilirubin (*r* = 0,257; *p* < 0,001) and negative with albumin (*r* = −0,265; *p* < 0,001) and PSH (*r* = −0,124; *p* < 0, 05) were observed. Results are shown in Table 3.

### 3.4. Relationships between Redox Parameters in Heart Failure due to Ischaemic and Nonischaemic Cardiomyopathy

#### 3.4.1. ICM

An inverse correlation between TAC and TOS (*p* = −0,139; *p* < 0, 01), OSI (*r* = −0,474; *p* < 0,001), and PSH (*r* = −0,265; *p* < 0,001) and a positive correlation with uric acid (*r* = 0,324; *p* < 0,001), MDA (*r* = 0,204; *p* < 0,001), the MDA/PSH index (*r* = 0,292; *p* < 0,001), and albumin (*r* = 0,102; *p* < 0,001) were found. MDA correlated positively with uric acid (*r* = 0,120; *p* < 0, 05) concentration and negatively with PSH (*r* = 0,207; *p* < 0,001). Additionally, uric acid positively correlated with albumin (*r* = 0,146; *p* < 0,001), bilirubin (*r* = 0,164; *p* < 0,001), and the MDA/SH index (*r* = 0,115; *p* < 0, 05) and inversely with the OSI index (*r* = −0,150; *p* < 0,001). Additionally, PSH correlated positively with OSI (*r* = 0,099; *p* < 0, 05) and albumin (*r* = 0,096; *p* < 0, 05). The results are shown in Table 4.

#### 3.4.2. nICM

Similarly to the ICM group, positive correlations between TAC and uric acid (*r* = 0,546; *p* < 0,001), bilirubin (*r* = 0,195; *p* < 0,001), MDA (*r* = 0,278; *p* < 0,001), and MDA/PSH (*r* = 0,366; *p* < 0,001) were found and negative correlations were found between TAC and SH (*r* = −0,267; *p* < 0,001) and OSI (*r* = −0,477; *p* < 0,001). In contrast to the ICM group, the correlation between TAC and TOS was not significant. MDA additionally correlates positively with bilirubin (*r* = 0,205; *p* < 0,001) and negatively with SH (*r* = −0,230; *p* < 0,001). A positive correlation between uric acid and TAC (*r* = 0,546; *p* < 0,001), MDA/PSH (*r* = 0,145; *p* = 0,016), and bilirubin (*r* = 0,151; *p* < 0, 01) was found. A negative correlation was found between uric acid and PSH (*r* = −0,178; *p* < 0, 01) and OSI (*r* = −0,191; *p* < 0,001).

Additionally, bilirubin correlated positively with MDA/PSH (*r* = 0,250; *p* < 0,001) and negatively with PSH (*r* = −0,179; *p* = 0,003). Results are shown in Table 5.

## 4. Discussion

To our knowledge, this is one of few studies assessing the oxidative stress index (OSI) as a TAC to TOS ratio, the products of lipid peroxidation, and the protein sulfhydryl group PSH in relation to heart failure due to ischaemic and nonischaemic aetiologies.

Additionally, we focused on the differences between redox-state compounds in the ischaemic and nonischaemic groups depending on the patient's clinical status and therapy.

In the present study, we evaluated a large group of 479 patients with ischaemic heart failure and 295 patients with nonischaemic heart failure. It should be emphasised that the patients were clinically stable and they received the optimal treatment for heart failure.

We have found significantly higher TAC concentrations in ischaemic patients which was accompanied by a significant decrease in OSI. In our study, TOS was similar in ischaemic and nonischaemic patients. Additional studies compared ischaemic or nonischaemic patients with a control group. Karabacak et al. studied 37 patients with nonischaemic HF. They observed that OSI levels were significantly higher in the patients with nonischaemic HF compared to the control subjects; uric acid and the TOS level were also higher in nonischaemic HF patients compared to the control group [23]. Animal studies by Hill and Singal demonstrated that HF subsequent to myocardial infarction was associated with an antioxidant capacity deficit as well as with increased oxidative stress [24]. Results obtained by Ellidag et al. showed that oxidative stress markers (TOS, OSI) were increased in patients with chronic ischaemic heart failure compared with healthy patients, whereas albumin and TAS levels were significantly lower in the chronic ischaemic heart failure patients [25]. Our results showing no differences in TOS between ischaemic and nonischaemic patients in terms of oxidative stress were convergent with the observations by Asoglu et al. [26]. Conversely, they observed a decrease in TAC in ischaemic patients comparing with the nonischaemic group. Surprisingly, in our ischaemic patients, TAC was significantly higher than that in the nonischaemic patients.

In our study, ICM patients were statistically older and had higher incidences of hypertension or diabetes. In addition, this group was characterised by higher fasting glycaemia, higher creatinine, and a lower haemoglobin level. The aforementioned differences may be partially explained by metabolic disorders, leading to the development and propagation of the atherosclerotic process at the base of ischaemic HF.

Both groups were comparable in terms of values of parameters such as LVEF, NT-proBNP concentration, and NYHA functional class. However, lower maximal minute oxygen consumption was indicated in the ICM group. An impaired metabolic profile and lower haemoglobin level may explain the lower peak of oxygen consumption in ICM. The concentration of uric acid and lipids was similar in both groups, but it may be the effect of more frequent use of allopurinol in nICM groups and statins in the ICM group. Referring to the clinical state of patients, both bilirubin and albumin correlated with the exercise tolerance of patients assessed by NYHA or cardiopulmonary exercise testing and NTproBNP in both groups. However, UA, especially in patients not treated with allopurinol, showed a positive correlation only to the ICM group.

Moreover, significantly lower PSH and a higher MDA concentration and MDA/SH ratio were found in the ICM group. It is very important to highlight that a negative correlation between the maximum oxygen consumption and the MDA concentration and a positive correlation with PSH were indicated. In addition, the concentration of MDA in both subgroups positively correlated with the NYHA functional class, while in the nICM group, it correlated negatively with PSH.

At the same time, there was no correlation between the LVEF and oxidative stress parameters such as PSH, MDA, TAC, TAS, OSI, and the MDA/SH ratio, in both groups. The association of LVEF with potential antioxidant activity compounds such as UA, bilirubin, and albumin has been demonstrated only in the group of patients with ICM. This supports the hypothesis that the reduction of LVEF is less important for oxidative stress generation than tissue hypoxia and neurohormonal activation as consequence of low cardiac output and maybe atherosclerotic lesions. The results obtained confirm the previous observation that disturbances of reduction and oxidative balance in patients with systolic heart failure are rather a result of heart failure than its cause [9].

Our results are partially consistent with data presented by Keith et al., who reported a correlation between the severity of heart failure and elevated lipid peroxides and MDA in both ischaemic heart disease and dilated cardiomyopathy patients with end-stage heart failure [27]. Similar results were obtained by McMurray et al. [28]. An elevated MDA concentration was measured in the plasma of patients with moderate symptoms of congestive heart failure. It has been compared to an age-matched control group. Additionally, we observed that PSH in ischaemic patients was significantly lower than that in the nonischaemic group. More interestingly, there was an inverse correlation between MDA and PSH in both the ischaemic and nonischaemic groups. Belch et al. and Radovanovic et al. demonstrated an increase in MDA and a decrease in PSH in HF patients compared to the control group [29, 30]. It is well-established that sulfhydryl groups of plasma proteins might act as “sacrificial” antioxidants in the plasma and extravascular spaces by intercepting chain reactions of radical production in plasma [30]. From the serum proteins, albumin is the most abundant thiol [31]. Other thiol-containing proteins, such as metallothionein, have significant antioxidant functions through direct scavenging of ROS and may play a role in the failing heart. PSH groups break the lipid peroxidation reaction with peroxyl radicals during the very first steps of the oxidation reaction [29, 30]. A significantly high concentration of plasma MDA and reduced thiol have also been reported by Levine et al. in heart failure patients with underlying coronary artery disease [32]. On the contrary, the study by Poildori et al. showed no differences in MDA concentration between patients with CHF of ischaemic and nonischaemic origins [33]. A negative correlation between PSH and MDA, on the one hand, may support the use of PSH in reactions that inhibit the growing process of lipid peroxidation or their primary deficiency. On the other hand, a negative correlation between PSH and MDA may support the use of PSH in reactions that inhibit the growing process of lipid peroxidation or their primary deficiency. Surprisingly, our study has shown a negative correlation between TAC and PSH and a positive one between TAC and MDA.

In ICM patients, MDA correlated with uric acid concentration positively too. A negative correlation between PSH and the concentration of uric acid and bilirubin was expressed only in the nICM group. This may be in favour of PSH consumption in the ROS inactivation reaction generated by the xanthine oxidase reaction and in the cyclic bilirubin oxidation to biliverdin reaction. The nICM and ICM groups are also different in terms of the degree of correlation between MDA and uric acid and bilirubin. In the ICM group, a significantly positive correlation occurs between uric acid and MDA, while in the nICM group, MDA correlates with uric acid and bilirubin. This suggests that in patients with ischaemic HF, the oxidation of purines to uric acid is a dominant source of ROS. In patients with nICM, ROS comes from reactions associated with heme degradation. The increase in uric acid levels under oxidative stress, as well as its reducing properties in vitro and in vivo, is well documented.

When analysing the relationship between UA and TAC, a positive correlation was found in both groups but it was stronger in nICM. Interestingly, both in ICM and in nICM, stronger correlations were demonstrated for patients treated with allopurinol.

Strong positive correlations between TAC and uric acid are not surprising, since these small molecules (like albumin, vitamin E, and vitamin C) are TAC components [34, 35].

It is possible that the plasma level of UA (also a significant component of TAC) represents an adaptive response to protect an organism against oxidative stress [36]. However, the interpretation that this is a positive response of the organism to an increase of oxidative potential may be too far reaching, as documented by numerous clinical observations [37, 38]. Hyperuricemia has been suggested to reflect the raised level of xanthine oxidase activity (XO) in HF [39]. The UA levels may reflect the activity of circulating XO, an important source of oxygen free radicals [40].

Fabbrini et al. showed, in an “in vivo” experiment, that total uric acid degradation by recombinant urate oxidase in insulin-resistant patients with elevated UA concentration caused a 45-95% decrease in TAC, with a parallel increase of muscle and systemic markers of oxidative stress by 25-40% [41]. The authors concluded that circulating UA is the major antioxidant and might help to protect against free radical oxidative damage. However, hyperuricemia was associated with haemodynamic abnormalities in nICM cardiomyopathy [42]. Despite the negative correlation of uric acid with OSI, a positive association with the increase in lipid peroxidation was observed only in ICM patients not treated with allopurinol. This suggests that the use of a xanthine oxidase inhibitor may have an additional beneficial effect on heart failure due to ischaemic cardiomyopathy.

Preliminary results of the study [43] showed a beneficial effect on the inhibition of xanthine oxidase activity on myocardial contractility and vascular endothelial function.

Unfortunately, large clinical trials have not demonstrated the benefits of inhibiting xanthine oxidase activity with allopurinol in patients with heart failure [44, 45]. That might be the question of patient selection. There are few reports in which a high plasma UA level, partly secreted from the failing heart, is a prognostic predictor, independent of BNP, in patients with CHF. Combination of BNP and UA monitoring may be useful for the management of patients with CHF. The senior's trial showed that UA is an independent predictor of death, both in systolic and in diastolic HF [46].

Kittleson et al. showed that serum uric acid levels correlate with plasma NT-proBNP and are associated with worse haemodynamic function in patients with HF [47]. In our study, the positive correlation between uric acid and NT-proBNP was observed in ICM and nICM patients, but only when they were not treated by allopurinol.

The study by Anker et al. documents and validates that high serum UA levels are strong, independent markers of a worse prognosis in patients with moderate to severe CHF [48]. Therefore, the most direct interpretation is that serum uric acid levels might not have a causal role in HF but rather represent a marker of an adverse prognosis, indicative of oxidative damage within the myocardium and/or the vasculature.

Our research suggests that bilirubin has similar properties to UA in the ischaemic group HF patient observation. The positive correlations between bilirubin and NT-proBNP and uric acid were observed. Higher bilirubin and lower albumin concentrations were associated with an increased severity of heart failure both in ICM and in nICM patients. These results, on the one hand, are likely to reflect the impairment of liver function in advanced heart failure. On the other hand, the demonstrated correlations between bilirubin and SH (negative) and MDA (positive) concentrations suggest that they are related to the oxidation reduction balance. Interestingly, these relationships only occur in the nICM patients. Is difficult to explain that the lipid peroxidation process is connected with higher bilirubin in nICM, in contrast to higher UA in the ICM group. The facts that bilirubin and uric acid correlated positively with TAC and that increased bilirubin or uric acid concentrations are related to adverse outcomes make it difficult to view these parameters as beneficial antioxidants [48, 49]. The results obtained are consistent with the results of Chuang et al. [50].

It seems that TAC is not protective in the literal sense. We cannot interpret the increase in TAC as a super defensive mechanism against ROS action in patients with heart failure, because of the fact that there were positive correlations between TAC and MDA in both groups.

We emphasise that the negative correlation between the final products of lipid peroxidation—MDA and PSH—were detected in both examined groups. In heart failure patients, Koning et al. showed that the concentration of PSH groups above the mean, was associated with better renal function, lower levels of NT-proBNP, and with a decreased rehospitalisation rate and increased patient survival [8].

These complement the results obtained by us. The presented study showed a positive correlation between the magnitude of maximum oxygen consumption and the concentration of SH groups. We also detected a negative correlation between PSH and NT-proBNP and the NYHA class in n-ICM patients. The negative correlation between TAC and PSH (part of TAC) speaks over the active growth of TAS components generated in oxidation reactions (uric acid) and consumption of others such as PSH, perhaps in a reaction with ROS generated in the synthesis of TAC components.

PSH negatively correlates with the concentration of uric acid and bilirubin. This is particularly expressed in the nICM group. This may be in favour of PSH consumption in the ROS inactivation reaction generated by the xanthine oxidase reaction and in the cyclic bilirubin oxidation to biliverdin reaction.

## 5. Conclusion

An important advantage of the study is the large size of the study group. In both groups of patients, we have demonstrated the relationship between the heart failure aetiology and oxidative stress. These findings are in favour of the dominant concept of the role of oxidative stress in CHF in the last decade [51, 52]. When comparing oxidative stress parameters in ICM and nICM patients and when analysing the correlations between oxidative stress parameters and clinical figures, it seems that the defence mechanisms against ROS are different for the ICM and the nICM groups.

## Figures and Tables

**Table 1:** Clinical and laboratory characteristics of patients included into the study with comparison of study groups. Categorical variables are shown as percentages and numerical variables as medians with lower and upper quartiles where appropriate.

	Ischaemic (*n* = 479)	Nonischaemic (*n* = 295)	*p* value
General characteristics			
Male (*n*) (%)	416 (86,8)	249 (84,4)	NS
Age (years)	56,00 (52,00–61,00)	50,00 (39,00–56,00)	0,001
BMI (kg/m^2^)	26,45 (23,88-29,29)	25,77 (22,32-29,07)	0,01
Duration of symptoms before inclusion (months)	33,87 (13,6-66,27)	33,83 (2,17-73,1)	NS
NYHA class	3 (2-3)	3 (2-3)	NS
Measured VO2 (ml/min)	1,10 (0,82-1,38)	1,16 (0,92-1,43)	0,05
Maximum measured VO2 (ml/min/kg b.m.)	14,6 (11,9-17,9)	15,6 (12,6-19,5)	0,01
Measured VCO2 (l/min)	1,07 (0,77-1,38)	1,13 (0,86-1,39)	0,01
LVEDD (mm)	69,00 (63,00-75,00)	71,00 (64,00-78,00)	0,05
LVEDV (ml)	209,0 (167,0-271,0)	245,0 (181,0-314,0)	0,001
LVEF (%)	24,00 (20,00-30,00)	23,00 (20,00-29,00)	NS
Basic biochemistry			
Haemoglobin (mmol/l)	14,02 (13,05-14,83)	14,18 (13,05-15,31)	0,05
Creatinine (*μ*mol/l)	88,00 (73,00–108,0)	83,00 (71,0–99,0)	0,01
Serum protein concentration (g/l)	71,00 (67,00–75,00)	71,0 (67,00–76,00)	NS
Albumin (g/l)	42,00 (39, 00–44,00)	42,00 (39,00–45,00)	NS
Fasting glucose (mmol/l)	5,60 (5,00-6,40)	5,50 (4,90-6,10)	0,05
Total cholesterol (mmol/l)	4,26 (3,65-5,23)	4,32 (3,62-5,19)	NS
Triglycerides (mmol/l)	1,21 (0,88-1,74)	1,21 (0,91-1,71)	NS
Cholesterol HDL (mmol/l)	1,16 (0,95-1,41)	1,14 (0,91-1,40)	NS
Cholesterol LDL (mmol/l)	2,43 (1,87 - 3,16)	2,48 (1,94-3,19)	NS
NT-proBNP (pg/ml)	1317 (628,4-2863)	1601 (658,6-3562)	NS
Comorbidities			
Diabetes (*n*) (%)	162 (33,8)	57 (19,3)	0,001
Arterial hypertension (*n*) (%)	287 (59,9)	136 (46,1)	0,001
Atrial fibrillation (*n*) (%)	85 (17,7)	98 (33,2)	0,001
ICD presence (*n*) (%)	75 (15,7)	47 (15,9)	NS
Smoker (*n*) (%)	361 (75,4)	202 (68,5)	0,05
Pharmacotherapy			
Beta-blockers (*n*) (%)	472 (98,5)	287 (97,3)	NS
ACE inhibitors (*n*) (%)	417 (87,2)	251 (85,1)	NS
Angiotensin-2 receptor blockers (*n*) (%)	48 (10,0)	34 (11,5)	NS
Loop diuretics (*n*) (%)	403 (84,1)	276 (93,6)	0,05
Thiazide diuretics (*n*) (%)	52 (10,9)	47 (15,9)	0,05
Aldosterone receptor antagonist (*n*) (%)	433 (90,4)	281 (95,3)	NS
Statins (*n*) (%)	364 (76,0)	142 (48,1)	0,001
Fibrates (*n*) (%)	17 (3,55)	11 (3,73)	NS
Digitalis (*n*) (%)	193 (40,3)	159 (53,9)	0,001
XOi (*n*) (%)	142 (29,6)	133 (45,1)	0,001

BMI: body mass index; NYHA: New York Heart Association functional class; VO2: maximum oxygen output; LVEDD: left ventricle end-diastolic diameter; LVEDV: left ventricle end-diastolic volume; LVEF: left ventricle ejection fraction; NT-proBNP: N-terminal pro-B-type natriuretic peptide; ICD: implantable cardioverter defibrillator; ACE-inhibitor: angiotensin-converting enzyme inhibitor; XOi: xanthine oxidase inhibitors; NS: nonsignificant.

**Table 2:** Redox biomarkers of patients included into a study with comparison of study groups. Variables are shown as medians with lower and upper quartiles where appropriate.

	Ischaemic (*n* = 479)	Nonischaemic (*n* = 295)	*p* value
TAC (mmol/l)	1,14 (1,04-1,26)	1,11 (0,98-1,22)	0,001
TOS (mmol/l)	4,75 (4-6,1)	5,10 (4,3-6,2)	NS
OSI (TOS/TAC)	4,27 (3,31-5,65)	4,60 (3,68-5,89)	0,01
Uric acid (*μ*mol/l)	407,0 (333,0–505,0)	413,0 (326,0-506,0)	NS
Uric acid (*μ*mol/l) XOi (-)	406,0 (336,0–491,0)	423,5 (344,5–497,5)	NS
Uric acid (*μ*mol/l) XOi (+)	410,5 (324,0–541,0)	403,0 (308,0–519,0)	NS
Bilirubin (*μ*mol/l)	12,70 (9,20-19,10)	15,40 (10,60-21,90)	0,001
Albumin (g/l)	42,00 (39,00–44,00)	42,00 (39,00–45,00)	NS
PSH (*μ*mol/g of protein)	4,10 (3,1-5,1)	4,75 (3,65-5,6)	0,001
MDA (*μ*mol/l)	1,80 (1,40-2,20)	1,70 (1,40-2,10)	0,05
MDA/PSH ratio	0,435 (0,314-0,647)	0,358 (0,269–0,527)	0,001

TAC: total antioxidant capacity; TOS: total oxidant status; OSI: oxidative stress index; MDA: malondialdehyde; PSH: sulfhydryl groups; XOi: xanthine oxidase inhibitors; NS: nonsignificant.

**Table 3:** Correlation between redox parameters and clinical characteristic of heart failure in ischaemic and nonischaemic cardiomyopathy.

	ICM	nICM
	Spearman *r*; *p*	Spearman *r*; *p*
	NYHA	NYHA
PSH (*μ*mol/g of protein)	*r* = 0,036; NS	*r* = −0,169; *p* < 0, 01
TAC (mmol/l)	*r* = 0,009; NS	*r* = 0,110; NS
TOS (mmol/l)	*r* = 0,110; *p* < 0, 05	*r* = 0,156; *p* < 0, 01
OSI (TOS/TAC)	*r* = 0,087; NS	*r* = 0,080; NS
MDA (*μ*mol/l)	*r* = 0,111; *p* < 0, 05	*r* = 0,152; *p* < 0, 05
MDA/PSH ratio	*r* = 0,063; NS	*r* = 0,225; *p* < 0,001
Uric acid (*μ*mol/l) (all)	*r* = 0,198; *p* < 0,001	*r* = 0,085; NS
Uric acid (*μ*mol/l) XOi (-)	*r* = 0,225; *p* < 0,001	*r* = 0,055; NS
Uric acid (*μ*mol/l) XOi (+)	*r* = 0,156; NS	*r* = 0,137; NS
Bilirubin (*μ*mol/l)	*r* = 0,269; *p* < 0,001	*r* = 0,244; *p* < 0,001
Albumin (g/l)	*r* = −0,095; *p* < 0, 05	*r* = −0,275; *p* < 0,001

	LVEF	LVEF
PSH (*μ*mol/g of protein)	*r* = −0,073; NS	*r* = −0,005; NS
TAC (mmol/l)	*r* = 0,036; NS	*r* = 0,046; NS
TOS (mmol/l)	*r* = −0,030; NS	*r* = −0,017; NS
OSI (TOS/TAC)	*r* = −0,031; NS	*r* = −0,012; NS
MDA (*μ*mol/l)	*r* = −0,079; NS	*r* = 0,100; NS
MDA/PSH ratio	*r* = −0,013; NS	*r* = 0,062; NS
Uric acid (*μ*mol/l) (all)	*r* = −0,176; *p* < 0,001	*r* = 0,002; NS
Uric acid (*μ*mol/l) XOi (-)	*r* = −0,240; *p* < 0,001	*r* = −0,118; NS
Uric acid (*μ*mol/l) XOi (+)	*r* = −0,025; NS	*r* = 0,092; NS
Bilirubin (*μ*mol/l)	*r* = −0,291; *p* < 0,001	*r* = −0,134; *p* < 0, 05
Albumin (g/l)	*r* = 0,090; *p* < 0, 05	*r* = 0,095; NS

	NT-proBNP	NT-proBNP
PSH (*μ*mol/g of protein)	*r* = −0,049; NS	*r* = −0,124; *p* < 0, 05
TAC (mmol/l)	*r* = −0,026; NS	*r* = 0,053; NS
TOS (mmol/l)	*r* = 0,048; NS	*r* = 0,104; NS
OSI (TOS/TAC)	*r* = 0,032; NS	*r* = 0,053; NS
MDA (*μ*mol/l)	*r* = 0,081; NS	*r* = −0,001; NS
MDA/PSH ratio	*r* = 0,102; *p* < 0, 05	*r* = 0,077; NS
Uric acid (*μ*mol/l) (all)	*r* = 0,172; *p* < 0,001	*r* = 0,025; NS
Uric acid (*μ*mol/l) XOi (-)	*r* = 0,214; *p* < 0,001	*r* = 0,183; *p* < 0, 05
Uric acid (*μ*mol/l) XOi (+)	*r* = 0,099; NS	*r* = −0,109; NS
Bilirubin (*μ*mol/l)	*r* = 0,328; *p* < 0,001	*r* = 0,257; *p* < 0,001
Albumin (g/l)	*r* = −0,275; *p* < 0,001	*r* = −0,265; *p* < 0,001

	VO2 maks.	VO2 maks.
PSH (*μ*mol/g of protein)	*r* = 0,084; NS	*r* = 0,179; *p* < 0, 01
TAC (mmol/l)	*r* = −0,049; NS	*r* = −0,114; NS
TOS (mmol/l)	*r* = −0,056; NS	*r* = 0,076; NS
OSI (TOS/TAC)	*r* = −0,013; NS	*r* = 0,119; NS
MDA (*μ*mol/l)	*r* = −0,129; *p* < 0, 05	*r* = −0,188; *p* < 0, 01
MDA/PSH ratio	*r* = −0,144; *p* < 0, 01	*r* = −0,216; *p* < 0,001
Uric acid (*μ*mol/l) (all)	*r* = −0,174; *p* < 0,001	*r* = −0,001; NS
Uric acid (*μ*mol/l) XOi (-)	*r* = −0,201; *p* < 0,001	*r* = 0,001; NS
Uric acid (*μ*mol/l) XOi (+)	*r* = −0,154; NS	*r* = −0,012; NS
Bilirubin (*μ*mol/l)	*r* = −0,262; *p* < 0,001	*r* = −0,314; *p* < 0,001
Albumin (g/l)	*r* = 0,131; *p* < 0, 01	*r* = 0,144; *p* < 0, 05

ICM: ischaemic cardiomyopathy; nICM: nonischaemic cardiomyopathy; NYHA: New York Heart Association functional class; LVEF: left ventricle ejection fraction; NT-proBNP: N-terminal pro-B-type natriuretic peptide; VO2max: maximum oxygen output; TAC: total antioxidant capacity; TOS: total oxidant status; OSI: oxidative stress index; MDA: malondialdehyde; PSH: sulfhydryl groups; XOi: xanthine oxidase inhibitors.

**Table 4:** Correlation between redox parameters in ischaemic heart failure patients.

ICM	PSH (*μ*mol/g)	TAC (mmol/l)	TOS (mmol/l)	OSI (TOS/TAC)	MDA (*μ*mol/l)	MDA/SH	Uric acid (*μ*mol/l)	Uric acid (XOi -) (*μ*mol/l) (*n* = 337)	Uric acid (XOi +) (*μ*mol/l) (*n* = 142)	Bilirubin (*μ*mol/l)	Albumin (g/l)
PSH (*μ*mol/lg)	1,000	-0,265 (*p* < 0,001)	-0,012 NS	0,099 (*p* < 0, 05)	-0,207 (*p* < 0,001)		-0,059 (NS)	-0,086 (NS)	-0,016 (NS)	-0,049 (NS)	0,096 (*p* < 0, 05)
TAC (mmol/l)	-0,265 (*p* < 0,001)		-0,139 (*p* < 0, 05)		0,204 (*p* < 0,001)	0,292 (*p* < 0,001)	0,324 (*p* < 0,001)	0,282 (*p* < 0,001)	0,399 (*p* < 0,001)	0,004 (NS)	0,102 (*p* < 0,001)
TOS (mmol/l)	-0,012 (NS)	-0,139 (*p* < 0, 05)			0,032 (NS)	0,062 (NS)	-0,025 (NS)	-0,054 (NS)	0,040 (NS)	-0,052 (NS)	-0,065 (NS)
OSI (TOS/TAC)	0,099 (*p* < 0, 05)				-0,072 (NS)	-0,078 (NS)	-0,150 (*p* < 0,001)	-0,162 (*p* < 0, 05)	-0,125 (NS)	-0,056 (NS)	-0,081 (NS)
MDA (*μ*mol/l)	-0,207 (*p* < 0,001)	0,204 (*p* < 0,001)	0,032 (NS)	-0,072 (NS)			0,120 (*p* < 0, 05)	0,125 (*p* < 0, 05)	0,105 (NS)	0,078 (NS)	0,060 (NS)
MDA/PSH ratio		0,292 (*p* < 0,001)	0,062 (NS)	-0,078 (NS)			0,115 (*p* < 0, 05)	0,143 (*p* < 0, 05)	0,066 (NS)	0,081 (NS)	-0,043 (NS)
Uric acid (*μ*mol/l)	-0,059 (NS)	0,324 (*p* < 0,001)	-0,025 (NS)	-0,150 (*p* < 0,001)	0,120 (*p* < 0, 05)	0,115 (*p* < 0, 05)				0,164 (*p* < 0,001)	0,146 (*p* < 0,001)
Uric acid (XOi -) (*μ*mol/l)	-0,086 (NS)	0,282 (*p* < 0,000)	-0,054 (NS)	-0,162 (*p* < 0, 01)	0,125 (*p* < 0, 05)	0,143 (*p* < 0, 05)				0,191 (*p* < 0,001)	0,148 (*p* < 0, 01)
Uric acid (XOi +) (*μ*mol/l)	-0,016 (NS)	0,399 (*p* < 0,001)	0,040 (NS)	-0,125 (NS)	0,105 (NS)	0,066 (NS)				0,102 (NS)	0,124 (NS)
Bilirubin (*μ*mo/l)	-0,049 (NS)	0,004 (NS)	-0,052 (NS)	-0,056 (NS)	0,078 (NS)	0,081 (NS)	0,164 (*p* < 0,001)	0,191 (*p* < 0,001)	0,102 (NS)		0,009 (NS)
Albumin (g/l)	0,096 (*p* < 0, 05)	0,102 (*p* < 0,001)	-0,065 (NS)	-0,081 (NS)	0,060 (NS)	-0,043 (NS)	0,146 (*p* < 0,001)	0,148 (*p* < 0, 01)	0,124 (NS)	0,009 (NS)	

ICM: ischaemic cardiomyopathy; TAC: total antioxidant capacity; TOS: total oxidant status; OSI: oxidative stress index; MDA: malondialdehyde; PSH: sulfhydryl groups; XOi: xanthine oxidase inhibitors.

**Table 5:** Correlation between redox parameters in nonischaemic heart failure patients.

nICM	PSH (*μ*mol/g)	TAC (mmol/l)	TOS (mmol/l)	OSI (TPS/TAC)	MDA (*μ*mol/l)	MDA/SH	Uric acid (*μ*mol/l)	Uric acid (XOi -) (*μ*mol/l) (*n* = 152)	Uric acid (XOi +) (*μ*mol/l) (*n* = 143)	Bilirubin (*μ*mol/l)	Albumin (g/l)
PSH (*μ*mol/g)		-0,267 (*p* < 0,001)	-0,021 (NS)	0,078 (NS)	-0,230 (*p* < 0,001)		-0,178 (*p* < 0, 01)	-0,193 (*p* < 0, 05)	-0,194 (*p* < 0, 05)	-0,179 (*p* < 0, 01)	0,049 (NS)
TAC (mmol/l)	-0,267 (*p* < 0,001)		-0,087 (NS)		0,278 (*p* < 0,001)	0,366 (*p* < 0,001)	0,546 (*p* < 0,001)	0,383 (*p* < 0,001)	0,706 (*p* < 0,001)	0,195 (*p* < 0,001)	0,051 (NS)
TOS (mmol/l)	-0,021 (NS)	-0,087 (NS)			0,112 (NS)	0,081 (NS)	0,050 (NS)	0,149 (NS)	-0,040 (NS)	0,098 (NS)	0,015 (NS)
OSI (TOS/TAC)	0,078 (NS)				-0,017 (NS)	-0,072 (NS)	-0,191 (*p* < 0,001)	-0,062 (NS)	-0,307 (*p* < 0,001)	0,007 (NS)	0,013 (NS)
MDA (*μ*mol/l)	-0,230 (*p* < 0,001)	0,278 (*p* < 0,001)	0,112 (NS)	-0,017 (NS)			0,067 (NS)	0,013 (NS)	0,149 (NS)	0,205 (*p* < 0,001)	0,061 (NS)
MDA/PSH ratio		0,366 (*p* < 0,001)	0,081 (NS)	-0,072 (NS)			0,145 (*p* < 0, 05)	0,092 (NS)	0,232 (*p* < 0, 01)	0,250 (*p* < 0,001)	0,010 (NS)
Uric acid (*μ*mol/l)	-0,178 (*p* < 0, 01)	0,546 (*p* < 0,001)	0,050 (NS)	-0,191 (*p* < 0,001)	0,067 (NS)	0,145 (*p* < 0, 05)				0,151 (*p* < 0, 01)	0,028 (NS)
Uric acid (XOi -) (*μ*mol/l)	-0,193 (*p* < 0, 05)	0,383 (*p* < 0,001)	0,149 (NS)	-0,062 (NS)	0,013 (NS)	0,092 (NS)				0,092 (NS)	-0,046 (NS)
Uric acid (XOi +) (*μ*mol/l)	-0,194 (*p* < 0, 05)	0,706 (*p* < 0,001)	-0,040 (NS)	-0,307 (*p* < 0,001)	0,149 (NS)	0,232 (*p* < 0, 01)				0,165 (*p* < 0, 05)	0,089 (NS)
Bilirubin (*μ*mol/l)	-0,179 (*p* < 0, 01)	0,195 (*p* < 0,001)	0,098 (NS)	0,007 (NS)	0,205 (*p* < 0,001)	0,250 (*p* < 0,001)	0,151 (*p* < 0, 01)	0,092 (NS)	0,165 (*p* < 0, 05)		-0,022 (NS)
Albumin (g/l)	0,049 (NS)	0,051 (NS)	0,015 (NS)	0,013 (NS)	0,061 (NS)	0,010 (NS)	0,028 (NS)	-0,046 (NS)	0,089 (NS)	-0,022 (NS)	

nICM: nonischaemic cardiomyopathy; TAC: total antioxidant capacity; TOS: total oxidant status; OSI: oxidative stress index; MDA: malondialdehyde; PSH: sulfhydryl groups; XOi: xanthine oxidase inhibitors.

## Data Availability

The data used to support the findings of this study are available from the corresponding author upon request.
